# The Effects of Multi-Mode Monophasic Stimulation with Capacitive Discharge on the Facial Nerve Stimulation Reduction in Young Children with Cochlear Implants: Intraoperative Recordings

**DOI:** 10.3390/jcm12020534

**Published:** 2023-01-09

**Authors:** Fabiana Danieli, Miguel Angelo Hyppolito, Raabid Hussain, Michel Hoen, Chadlia Karoui, Ana Cláudia Mirândola Barbosa Reis

**Affiliations:** 1 Postgraduate Program at the Department of Health Sciences, RCS, Ribeirão Preto Medical School, University of São Paulo, Bandeirantes 3900, Ribeirão Preto 14049-900, Brazil; 2 Clinical Department, Oticon Medical, Lino de Moraes Leme 883, São Paulo 04360-001, Brazil; 3 Department of Ophthalmology, Otorhinolaryngology, Head and Neck Surgery, Ribeirão Preto Medical School, University of São Paulo, Bandeirantes 3900, Ribeirão Preto 14049-900, Brazil; 4 Research & Technology Department, Oticon Medical, 2765 Smørum, Denmark; 5 Clinical Evidence Department, Oticon Medical, 2720 Chem de Saint-Bernard, 06220 Vallauris, France; 6 Department of Health Sciences, RCS, Ribeirão Preto Medical School, University of São Paulo, Bandeirantes 3900, Ribeirão Preto 14049-900, Brazil

**Keywords:** facial nerve stimulation, cochlear implant, electromyography, stimulation strategy, image analysis, computed tomography, image segmentation, 3D model

## Abstract

Facial nerve stimulation (FNS) is a potential complication which may affect the auditory performance of children with cochlear implants (CIs). We carried out an exploratory prospective observational study to investigate the effects of the electrical stimulation pattern on FNS reduction in young children with CI. Ten ears of seven prelingually deafened children with ages up to 6 years old who undergone a unilateral or bilateral CI surgery were included in this study. Electromyographic (EMG) action potentials from orbicularis oculi muscle were recorded using monopolar biphasic stimulation (ST1) and multi-mode monophasic stimulation with capacitive discharge (ST2). Presence of EMG responses, facial nerve stimulation thresholds (T-FNS) and EMG amplitudes were compared between ST1 and ST2. Intra-cochlear electrodes placement, cochlear-nerve and electrode-nerve distances were also estimated to investigate their effects on EMG responses. The use of ST2 significantly reduced the presence of intraoperative EMG responses compared to ST1. Higher stimulation levels were required to elicit FNS with ST2, with smaller amplitudes, compared to ST1. No and weak correlation was observed between cochlea-nerve and electrode-nerve distances and EMG responses, respectively. ST2 may reduce FNS in young children with CI. Differently from the electrical stimulation pattern, the cochlea-nerve and electrode-nerve distances seem to have limited effects on FNS in this population.

## 1. Introduction

Cochlear implant (CI) is the most effective treatment option for young children with profound sensorineural hearing loss. A longitudinal study showed that children implanted up to 2 years old scored on average above 50% on open-set speech recognition tasks after 4 years of CI experience [1]. The rate of complications with cochlear implantation has also decreased with advances in the CI field in the last years [2]. However, one potential complication that persists and affects the auditory performance of children with CI is the facial nerve stimulation (FNS).

FNS incidence was reported to range from 1.14% to 43% in children, with immediate or delayed onset [3]. Although it is known that otosclerosis, meningitis, temporal bone fractures and congenital cochlear anomalies increase the risk of FNS, some patients experience it after cochlear implantation without any of these etiologies. FNS symptoms may range from mild facial movements to severe facial spasms, painful or debilitating [4], either visually detected or self-reported by the patient. In young children, FNS has been underestimated, as they may not accurately report its symptoms. Cushing, Papsin and Gordon [5] reported a large difference in the incidence of FNS in children with CI when electrophysiological recordings are compared to their reports or even to observation of facial movements.

It is assumed that the electric current passing from the electrode to spiral ganglion cell can spread to the nearby facial nerve causing FNS [6], but the exact mechanism underlying the FNS remains unclear, as well as the relative contribution of factors to trigger the symptoms and the best treatment option to resolve it. 

Some strategies have been adopted to manage FNS symptoms, including maximum comfort levels (MCL) reduction [7], pulse wide widening [8], the use of triphasic pulses [9], electrode deactivation [10] and cochlear re-implantation [11]. However, these strategies may result in auditory performance decline [7] and does not ensure to resolve FNS [8].

Recently, the use of the multi-mode monophasic stimulation was proposed as a promising strategy to manage FNS [7,12,13]. It resolved severe FNS in some adult CI recipients, after cochlear re-implantation with the Neuro Zti device (Oticon Medical, Smørum, Denmark). Most current CI devices use monopolar biphasic stimulation, and, in this CI electrical stimulation pattern, the total amount of electrical charge flows from intra-cochlear electrodes to extra-cochlear ground electrodes, and each phase of the pulse stimulates different group of neurons, increasing the spatial extent of stimulation. Using multi-mode monophasic stimulation with subsequent capacitive discharge, most of the electrical current is maintained within the cochlea, and the anodic stimulating phase of the monophasic pulse is followed by a non-stimulating cathodic phase (with reduced amplitude, compared to anodic phase). It is hypothesized that multi-mode monophasic stimulation decreases the spatial extent of electrical stimulation and reduces the amount of the current spread to the periphery structures, including the facial nerve, thereby reducing FNS. 

To the best of our knowledge, the effects of multi-mode monophasic stimulation on FNS reduction in children were not previously investigated. Thus, in this study, we recorded intraoperative EMG action potentials to investigate the use of the multi-mode monophasic stimulation in young children and the effects of this stimulation pattern on the FNS reduction in this population. We also used 3D image processing techniques to estimate the CI intra-cochlear electrodes placement, as well as the distances between the basal turn of the cochlea and electrodes (based on their real intra-cochlear positioning) to the labyrinthine segment of the facial nerve, to investigate their influence on the EMG recordings.

## 2. Materials and Methods

This was an exploratory prospective observational study approved by the Institutional Ethics Committee under protocol 5.117.640. Parental informed consent was obtained from all subjects involved in the study.

### 2.1. Subjects

Ten ears from seven prelingually deafened children aged up to 6 years old who undergone either unilateral or bilateral CI surgeries were included in this study. All subjects were implanted with the Neuro Zti Evo^®^ device associated to the Neuro 2 sound processor (Oticon Medical, Smørum, Denmark). The exclusion criteria was comprised of subjects with preoperative facial palsy or other facial nerve dysfunctions, neuromuscular diseases, and epilepsy, as they could affect the EMG responses. T demographic data of the subjects are shown in the Table 1. 

### 2.2. Procedure

EMG responses were recorded in all subjects during CI surgery, under general anesthesia (Propofol and Fentanyl). The duration of measurement was about 10–15 min to not prolong the surgery time, and it was carried out immediately after the insertion of the EVO^®^ electrode array inside the cochlea of the subjects. Pre-anesthetic sedatives were not administrated to avoid muscle relaxation and their influence on the EMG recordings and facial nerve monitoring.

FNS stimulation was investigated through the EMG action potentials recorded from the orbicular oculi or oris muscles, both innervated by the facial nerve. Prior to sterile surgical preparation, bipolar needle electrodes were placed inside the orbicularis oculi and oris muscles, ipsilateral to the cochlear implantation site. Intraoperative facial nerve monitoring was initially performed to assess EMG recordings from inputs (i.e., orbicularis oculi and oris muscles) using the Nerve Integrity Monitor—NIM-2 equipment (Medtronic Xomed Inc., Jacksonville, FL, USA). After insertion of the electrode array, the experimental protocol was performed firstly using only the orbicularis oculi input channel, to limit the duration of the measurement in young children. If absent or no clear responses were recorded from this channel, the orbicularis oris input channel was then used.

### 2.3. Stimulation Parameters and EMG Responses

The intracochlear electrical stimulation was produced by the cochlear implant, using the eCAP tool of the Genie Medical CI fitting software, version 1.6, and CI-Link interface (Oticon Medical, Smørum, Denmark), connected to a computer and external antenna, responsible for transmitting the electrical stimuli to internal antenna via radiofrequency. Four electrodes were tested in each ear: one basal (E1), two medial (E8, E15) and one apical (E20). For the facial nerve stimulation thresholds (T-FNS) investigation purposes, current levels started from 20 SA (stimulation amplitude, 1 SA = 1/45 mA) and increased by 5 SA steps until reach 70 SA (maximum current level). The pulse duration was fixed at 30 SD (stimulation duration, 1 SD = 1 µs). The stimulation level (nC/phase) at which an EMG response was first evoked was defined as T-FNS, and no further increase in stimulus level was performed after detection of an FNS response. Peak-to-peak amplitudes of EMG responses at T-FNS were also recorded.

**Figure 1:**
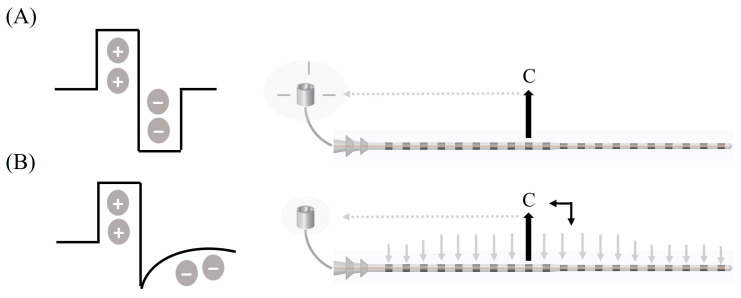
Schematic representation of different stimulation patterns used in this study. (**A**) Monopolar biphasic stimulation (stimulation pattern ST1) and (**B**) multi-mode monophasic stimulation with capacitive discharge (stimulation pattern ST2).

In order to investigate EMG responses using the two different CI stimulation patterns, the experimental protocol first employed monopolar biphasic stimulation (ST1) and then, multi-mode monophasic stimulation (ST2). Figure 1 shows the schematic of different stimulation patterns used in this study. Stimulation parameters used to record EMG responses are provided in the Table 2.

### 2.4. Radiological Examination

Pre- and postoperative CT scans of the ears were performed to investigate the intra-cochlear electrodes placement and the distance between the cochlea and labyrinthine segment of the facial nerve (cochlea-nerve distance). For this purpose, postoperative CT scans were performed in all subjects three months after cochlear implantation. 

**Figure 2:**
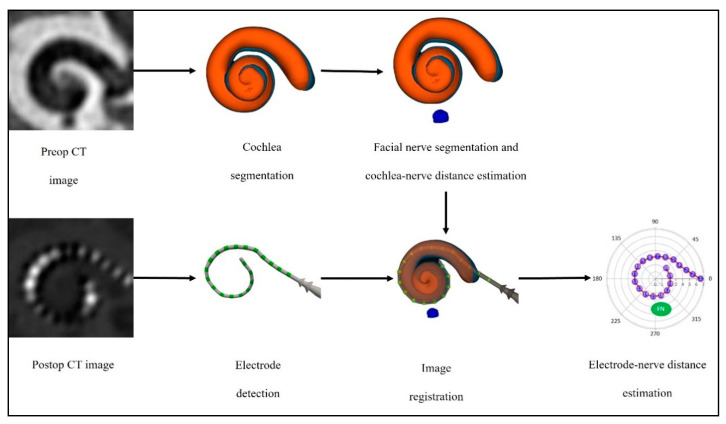
Model of CT image reconstruction performed by the software Nautilus in this study: cochlear segmentation, angular positioning of the electrodes and cochlear-nerve and electrode-nerve distances estimation.

The CT-scans were acquired at a resolution of 0.3 × 0.3 × 0.4 mm^3^. CT image reconstruction was performed using a web-based research platform Nautilus [14], which combines a convolutional neural network (CNN) approach with Bayesian joint appearance and shape inference for segmenting the cochlea and determining the trajectory of the electrode arrays. The angular positioning of the electrodes was determined automatically using Nautilus which extracts electrode position from postoperative CT-scan using a CNN approach and registers it with preoperative CT-scan for determining the electrode position with respect to the cochlear segmentation. The closest distance between the lateral wall of the cochlea and the labyrinthine segment of the facial nerve (cochlea-nerve distance) were measured on the pre-operative CT-scans. Next, the cochlea-nerve distance at each electrode’s angular location (electrode-nerve distance) were also measured to estimate the electrodes placed closest to the facial nerve. The 3D cochlear view reconstruction, including scala tympani segmentation, was input to Slicer 3D and manual annotation of the facial nerve was performed (Figure 2).

### 2.5. Statistical Analyses

The proportion of EMG responses with ST1 and ST2 were compared using the McNemar’s test. Spearman’s correlation test was used to investigate associations between intraoperative EMG responses and cochlea-nerve distances estimation of the subjects. The correlation between the electrode-nerve distances estimation of the electrode E15 (placed in the range closest to the facial nerve in most subjects, from 250 to 290 degrees) and T-FNS were also investigated. The results were expressed in correlation coefficient (ρ) and *p*-value. A significance level of 5% was adopted.

## 3. Results

Table 3 shows the proportion of intraoperative EMG responses recorded on each tested electrode using the stimulation patterns ST1 and ST2. ST1 stimulation leaded to intraoperative EMG responses in at least one electrode in 9 of 10 ears while the ST2 stimulation induced EMG responses only in the most basal electrode (E1) in 1 of 10 ears (#S4). Subject #S3 (EA6) showed absent EMG recordings with both stimulation patterns ST1 and ST2 (with orbicularis oculi and oris muscles input channels). Overall, the paired analyses indicated that the use of ST2 was significantly associated to lower incidence of intraoperative EMG responses recorded on all tested electrodes compared to ST1.

**Figure 3:**
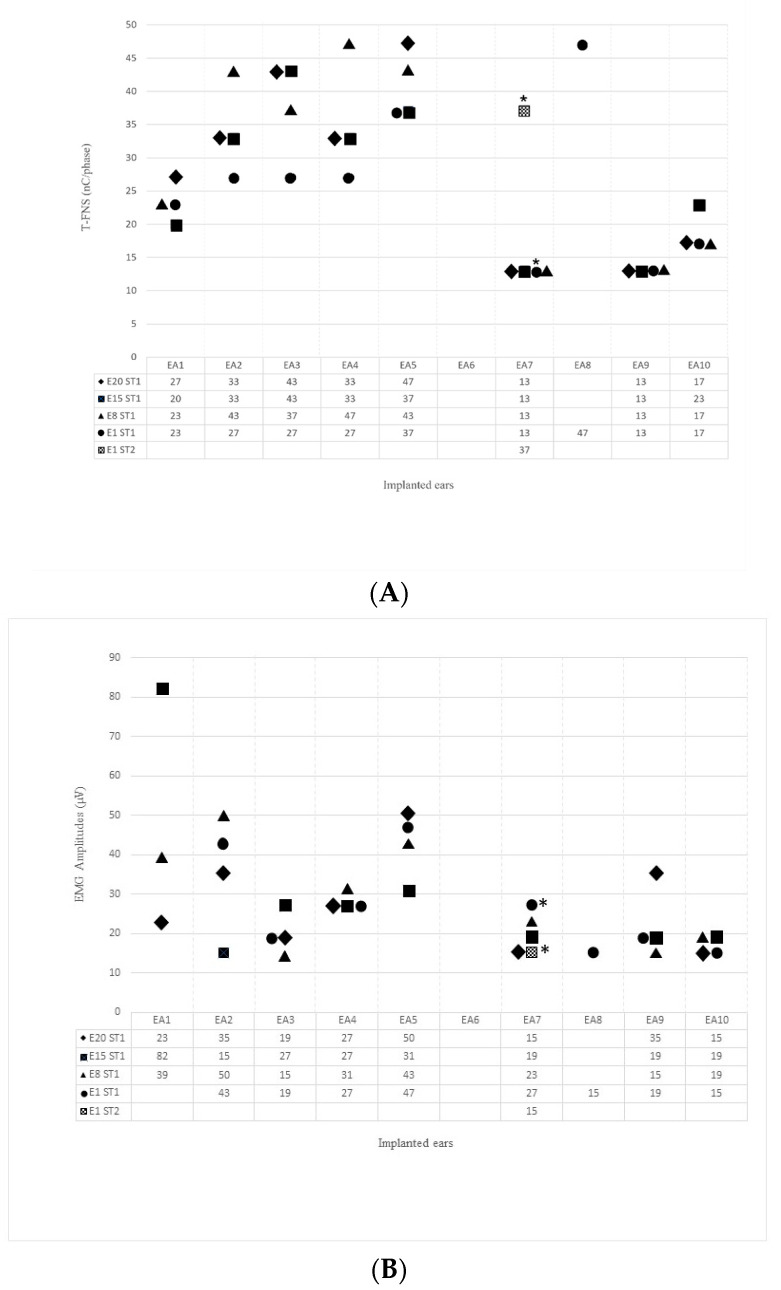
Individual T-FNS (**A**) and EMG amplitudes (**B**), when recorded, using CI stimulation patterns ST1 and ST2. Blank: no EMG responses. EA1–EA10: implanted ears 1–10. *Asterisks: comparison between values recorded with ST1 and ST2, in the same electrode (#Subject 4, EA7).

Figure 3 shows individual T-FNS (A) and EMG amplitudes (B), when recorded, using CI stimulation patterns ST1 and ST2. Two subjects showed EMG responses only in the electrode E1 (#S5 with ST1 and #S4 with ST2), and their absence in the remaining electrodes. Higher stimulation levels were required to elicit FNS (T-FNS) with ST2 (37 nC/phase) compared to ST1 (13 nC/phase) in the subject #S4 (electrode E1). Furthermore, peak-to-peak EMG amplitudes were smaller using ST2 (15 µV) compared to ST1 (27 µV) in this electrode.

**Figure 4:**
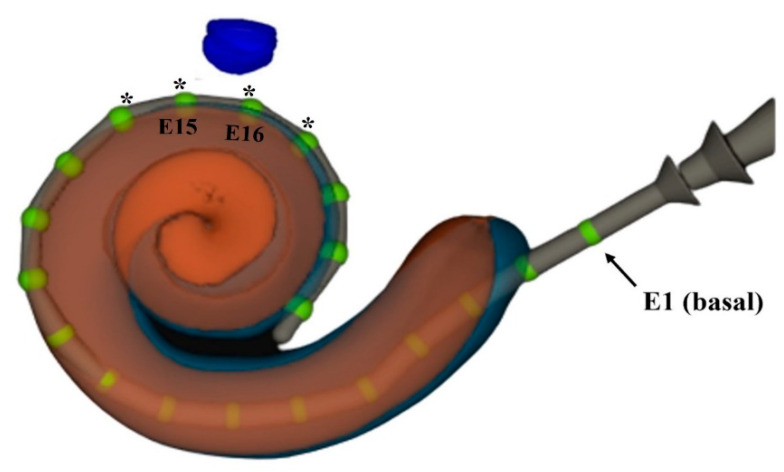
Example of electrodes placement estimation performed by Nautilus for the subject #S4 (EA7), who showed FNS responses with ST1 and ST2, in the electrode E1. Electrode 1 (basal) is extra-cochlear. * Blue circle and asterisks correspond to the labyrinthine segment of the facial nerve and Evo^®^ electrodes with closest electrode-nerve distances, respectively.

The intra-cochlear electrodes placement and cochlea-nerve distances estimation are provided in the Table 4. Four subjects (six ears) showed at least one extra-cochlear electrode placement (basal electrodes), including the subject #4, who showed EMG responses only in the most basal electrode with ST2 (Figure 4). The cochlea-nerve distances ranged from 0.20 to 1.00 mm (mean = 0.42 ± 0.26 mm). One subject (#S3, EA6) showed the longest cochlea-nerve distance and absent intraoperative EMG recordings with both the ST1 and ST2, but no correlation was observed between cochlear-nerve distances and EMG responses (Table 5). E15 and E16 were the Evo^®^ electrodes placed closest to the labyrinthine segment of the facial nerve, being recorded from 250 to 290 degrees insertion depth angle in most subjects. The electrode-nerve distance from the electrode E15 showed a weak correlation with the T-FNS recorded on this electrode (*ρ* = 0.42; *p*-value = 0.30). 

## 4. Discussion

The purpose of this study was to investigate the effects of the CI electrical stimulation pattern on FNS reduction in young children. We recorded intraoperative EMG action potentials in children implanted up to 6 years old, using two different CI electrical stimulation patterns: monopolar biphasic stimulation (ST1) and multi-mode monophasic stimulation with capacitive discharge (ST2). Presence of EMG responses, T-FNS and EMG amplitudes were compared between them. Multi-mode monophasic stimulation with capacitive discharge significantly reduced the presence of EMG responses compared to monopolar biphasic stimulation using equal stimulation levels in young children with CI. 

To the best of our knowledge, this is the first study comparing intraoperative EMG action potentials between ST1 and ST2 electrical stimulation patterns in CI children recipients and providing electrophysiological evidence of ST2 on FNS reduction in this population. Similar results were recently reported by Eitutis et al. [7] in three adult patients re-implanted with the Neuro Zti device due to severe FNS. Intraoperative FNS were recorded in all the three subjects with ST1, while no FNS was observed with ST2. In our study, FNS was recorded only in the most basal electrode of the subject #S4 with ST2, and the 3D image reconstruction revealed that this electrode was extra-cochlear (Table 4, Figure 4). It is known that extra-cochlear electrodes increase the risk of FNS in CI recipients [15,16], since the total amount of current spread from electrodes which lie outside the cochlea to the periphery structures, including the facial nerve. Thus, considering only the electrodes placed inside the cochlea and, therefore, excluding the extracochlear electrodes from the analysis, the FNS was not recorded with ST2 in any of the subjects included in this study. Our results reinforce those found by Eitutis et al. [7] in adults and suggest that the stimulation pattern ST2 seems to have comparable effects on FNS reduction in the first CI implantation of young children.

Higher stimulation levels were required to elicit FNS with ST2, with smaller EMG amplitudes, compared to ST1. This is also the first time that EMG input-output functions [9] (i.e., charge level required to elicit FNS versus EMG amplitude) could be compared between ST1 and ST2. The CI stimulation pattern ST2 corresponds to the combination of both, the multi-mode grounding stimulation and anodic monophasic pulse shape with capacitive discharge. The relative contribution of each of these features in FNS reducing in CI recipients has been unclear, since the software Genie Medical CI (Oticon Medical, Denmark) does not allow their dissociation for stimulation. In the multi-mode grounding stimulation, a greater amount of electrical current is maintained inside the cochlea since it flows from a stimulating intra-cochlear electrode to the remaining non-stimulating intra-cochlear electrodes. Considering that the electrode E1 (#S4) was extra-cochlear, the comparison of the EMG input-output functions between ST1 and ST2 seems to have been mainly influenced by the pulse shape, since the total amount of the electrical current was spread outside the cochlea, similarly to the monopolar stimulation mode pathway.

The distance between the basal turn of the cochlea and the labyrinthine segment of the facial nerve in the subjects ranged from 0.20 to 1.00 mm. These results are in accordance with previous studies on human temporal bones based on histological image measurements or macroscopical/microscopical analysis [17,18] and CI adult recipients based on pre-operative CT scans analysis (axial and coronal orientation plan) [4]. Hatch et al. [4] also investigated the effects of the cochlea-nerve distance on FNS in 49 ears of adult CI recipients and found lower cochlea-nerve distances in subjects with FNS compared to a control group (with no FNS). They suggested that cochlea-nerve distances longest than 0.6 mm should decrease the risk of FNS in this population. In our study, no correlation was observed between cochlear-nerve distances and intraoperative EMG responses in young children with CI, but one subject (#S3, EA6) showed cochlea-nerve distance longer than 0.6 mm, and only this subject showed absent intraoperative EMG recordings with both the ST1 and ST2. The Evo^®^ electrodes E15 and E16 were the closest to the labyrinthine segment of the facial nerve, and they were placed in the upper basal turn of the cochlea, from 250 to 290 degrees insertion depth angle, in most subjects (Figure 4). Seyyedi et al. [19] supposed that electrodes placed in the upper basal turn of cochlea should be most likely to excite the facial nerve, due to their proximity to it. Our findings may confirm the closest proximity of the electrodes placed in the upper basal turn of cochlea to the labyrinthine segment of the facial nerve, but only a weak correlation was observed between the electrode-nerve distance and intraoperative FNS responses, based on our results to the electrode E15. One limitation of this analysis was the small sample size (N = 10), nevertheless, strong correlations between these factors should be detected using the Spearman’s correlation test at *p* < 0.05. Even though, further investigations with a larger number of subjects are required to explore the results of intra-cochlear electrodes positioning to better understand the relative influence of this factor on FNS reduction in this population. Anyway, the use of 3D image processing techniques allowed us to accurately estimate the CI electrodes positioning and electrode-nerve distances based on the real intra-cochlear electrodes’ placement. This analysis was essential and provided better investigation on these aspects, considering the high variability in the CI electrodes positioning [20,21]. 

In this study, the EMG recordings were carried out under general anesthesia, and, as muscle relaxants could affect the EMG responses [9], they were not administrated, and thereby our findings may be compared to the clinical routine of non-anesthetized patients. 

Finally, our results suggest that CI electrical stimulation pattern may affect the FNS in young children and multi-mode monophasic stimulation with capacitive discharge should far reduce FNS in young children with CIs. The adoption of this electrical stimulation pattern should be an effective option for patients with a higher risk of experiencing FNS after CI surgery, such as patients with otosclerosis, meningitis, temporal bone fractures and congenital cochlear anomalies, or those who have indication for cochlear re-implantation due to severe FNS.

## 5. Conclusions

Multi-mode monophasic stimulation with capacitive discharge may reduce FNS in young children with CIs. Differently from the CI electrical stimulation pattern, the cochlea-nerve and electrode-nerve distances seem to have limited effects on FNS reduction in this population.

## Figures and Tables

**Table 1:** Subject demographics.

Subject	Ear	Side	Sex	Age at CI Surgery (m)	Etiology
S1	EA1	L	M	18	Congenital
	EA2	R			Congenital
S2	EA3	R	F	18	Genetics
	EA4	L			Genetics
S3	EA5	R	F	14	Idiopathic
	EA6	L			Idiopathic
S4	EA7	R	F	66	Ototoxicity
S5	EA8	L	F	47	Idiopathic
S6	EA9	R	F	45	Auditory neuropathy
S70	EA10	L	F	30	Genetics

S1–S7: subjects 1–7; EA1–EA10: implanted ears 1–10; L: left; R: right; M: male; F: female; CI: cochlear implant; m: months.

**Table 2:** Stimulation parameters used to record EMG responses.

Stimulation Patten	Stimulation Mode	Waveform	Polarity	Pulse Train	Coding	Pulse Amplitude Min:Step:Max (µA)	Pulse Duration (µs)	Stimulation Rate (Hz)
1 Monopolar biphasic	MP	Biphasic active symmetrical	Anodic leading	Masker probe	Amplitude	444:110:1554	30	83
2 Multi-mode Monophasic with CD	MM	Monophasic capacitive discharge	Anodic leading	Continuous	Amplitude	444:110:1554	30	250

CD: capacitive discharge; MP: monopolar; MM: multi-mode grounding, min: minimum; max: maximum.

**Table 3:** Proportion of intraoperative EMG responses recorded in each tested electrode using the stimulation patterns ST1 and ST2.

Electrode	ST1 N (%)	ST2 N (%)	*p*-Value
E1 (basal)	9 (90.0)	1 (10.0)	0.0143 *
E8 (medial)	8 (80.0)	0 (0.0)	0.0047 *
E15 (medial)	8 (80.0)	0 (0.0)	0.0047 *
E20 (apical)	8 (80.0)	0 (0.0)	0.0047 *

ST1: stimulation pattern 1 (monopolar biphasic stimulation); ST2: stimulation pattern 2 (multi-mode monophasic stimulation); N: number of EMG responses in each implanted ear. * Significant difference (McNemar’s test, 5% of significance level).

**Table 4:** Intra-cochlear electrodes placement, cochlea-nerve and electrode-nerve distances estimation of the subjects.

Subject	Ear	Side	Extra-Cochlear Electrodes	Cochlear-Nerve Distance (mm)	Insertion Depth Angle (°)	Electrode Closest to the FN
S1	EA1	L	0	0.24	279	15
	EA2	R	2	0.20	281	18
S2	EA3	R	1	0.20	285	15
	EA4	L	1	0.40	290	15
S3	EA5	R	1	0.44	267	16
	EA6	L	2	1.00	270	16
S4	EA7	R	1	0.20	273	16
S5	EA8	L	0	0.32	279	14
S6	EA9	R	0	0.56	276	11
S70	EA10	L	0	0.64	250	10

S1–S7: subjects 1–7; EA1–EA10: implanted ears 1–10; L: left; R: right; cochlea-nerve distance: closest distance between basal turn of the cochlea and the labyrinthine segment of the facial nerve; mm: millimeters; Electrode closest to the facial nerve: Evo^®^ electrode with closest electrode-nerve distance values; FN: facial nerve.

**Table 5:** Relationship between cochlear-nerve distances and EMG responses of the subjects.

	Spearman (rho)		*p*-Value	
Electrode	T-FNS (nC/Phase)	EMG Amplitude (µV)	T-FNS (nC/Phase)	EMG Amplitude (µV)
E20	−0.1975	0.1605	0.6391	0.7042
E15	−0.1975	0.0881	0.6391	0.8358
E8	−0.1605	−0.2332	0.7042	0.5784
E1	−0.1384	−0.2981	0.7439	0.4732

nC: Nanocoulomb; µV: microvolt; T-FNS: facial nerve stimulation thresholds; EMG Amplitude: peak-to-peak electromyographic amplitudes. Spearman’s correlation test, at a significant level of 5%.

## Data Availability

The data presented in this study are available on request from the corresponding author.
